# K_ATP_ Opener Attenuates Diabetic-Induced Müller Gliosis and Inflammation by Modulating Kir6.1 in Microglia

**DOI:** 10.1167/iovs.62.2.3

**Published:** 2021-02-01

**Authors:** Hong Li, Donglong Chen, Wei Sun, Jiansu Chen, Chang Luo, Heping Xu, Jacey Hongjie Ma, Shibo Tang

**Affiliations:** 1 AIER School of Ophthalmology, Central South University, Changsha, China; 2 AIER Eye Institute, Changsha, China; 3 Department of Ophthalmology, Guangdong Women and Children Hospital, Guangzhou, China; 4 CAS Center for Excellence in Brain Science and Intelligence Technology, Shanghai, China; 5 Centre for Experimental Medicine, School of medicine, Dentistry & Biomedical Science, Queen's University Belfast, Belfast, United Kingdom

**Keywords:** diabetic retinopathy, potassium channel, microglia, Müller cell, inflammation

## Abstract

**Purpose:**

This study aimed to determine the effect of pinacidil, a nonselective K_ATP_ channel opener, on diabetes-induced retinal gliosis and inflammation.

**Methods:**

Primary and immortalized cell lines of retinal microglia and Müller cells were used to set up a coculture model. In the trans-well system, microglia were seeded in the upper chamber and Müller cells in the bottom chamber. Microglia were polarized into proinflammatory (M1, with lipopolysaccharide and INF-γ) with or without different pinacidil concentrations before coculturing with Müller cells. The expression of inflammatory or anti-inflammatory genes and protein in microglia, and the expression of glial fibrillary acidic protein (GFAP), Kir4.1, and AQP4 in Müller cells were examined by real-time polymerase chain reaction and Western blot. Pinacidil was injected intravitreally into streptozotocin-induced diabetic rats. Retinal gliosis and inflammation were examined by immunohistochemistry and Western blot.

**Results:**

Intravitreal injection of pinacidil alleviated diabetes-induced Müller cell gliosis and microglial activation and reduced vascular endothelial growth factor expression. In vitro study demonstrated that pinacidil inhibited tumor necrosis factor and interleukin-1β expression in M1-type microglia and alleviated the M1 microglia-induced GFAP expression in the Müller cells. Furthermore, we found that pinacidil on its own, or in combination with IL-4, can upregulate arginase-1 (Arg-1) and Kir6.1 expression in microglial cells.

**Conclusions:**

Our results suggest that potassium channels are critically involved in diabetes-induced gliosis and microglial activation. The K_ATP_ opener, pinacidil, can reduce microglial activation by upregulating Kir6.1 expression.

Diabetic retinopathy (DR) is the most frequent sight-threatening complication in patients suffering from diabetes.^1^^,^^2^ Although it is well known that dysfunction and loss of retinal vasculature plays a central role in developing DR, the critical impact of the macroglial and microglial cells’ interaction on the vascular degeneration is gaining more and more attention.^3^ Overexpression of glial fibrillary acidic protein (GFAP), propermeability, and proinflammatory cytokines in diabetic rodent retinas, like VEGF, IL-6, and TNF-α were detected by different research groups.^3^^–^^5^

Microglial activation and translocation associated with the breakdown of the blood-retina-barrier were also demonstrated.^4^ Microglia can be induced into two activation phenotypes by different environmental factors, known as proinflammation subtype M1 and anti-inflammation subtype M2.^2^ Arginase 1 (Arg-1) is an enzyme that catalyzes L-arginine, acting as a competitor of the inducible isoform of nitric oxide synthase. As a result, L-ornithine is generated instead of nitric oxide (NO). NO favors nitrosative and oxidative stress and the M1 phenotype, whereas ornithine aminotransferase generates hydroxyproline, contributing to the maintenance of the extracellular matrix and reinforcing the M2 program.^5^^,^^6^

Inwardly rectifying K^+^ channel 4.1 (Kir4.1) and Aquaporin-4 (AQP4) are responsible for maintaining the water homeostasis and K^+^ buffering of the retina.^7^ Normally, Kir4.1 and AQP4 are overlapped in space and concentrated in the end-feet and perivascular processes of Müller cells,^8^^,^^9^ although this polarized expression of Kir4.1 is strongly reduced in experimental diabetes.^10^^,^^11^ Recent studies showed that the expression of Kir4.1 and AQP4 was altered in diabetic rat retina and may be associated with the development of diabetic macular edema (DME).^7^^,^^11^ Our previous investigations demonstrated that high glucose could disturb the expression of Kir4.1 and AQP4, IL-6, and VEGF in Müller cells, and these changes could be inverted by pinacidil.^12^ It has been suggested that pinacidil may be a potential intervention on diabetes-induced disturbance of water homeostasis and K^+^ buffering function. However, the pinacidil-mediated mechanism of the regulation of Kir4.1, and the non-ATP-dependent potassium channel remains elusive. It is noteworthy that an ATP-dependent potassium channel protein, Kir6.1, in brain microglia,^13^^,^^14^ has been shown to play a role in modulating microglia phenotypes in Parkinson's disease.^15^ The crosstalk between Müller cells and microglia is known to be able to drive neuroinflammation.^16^^,^^17^

In the present study, we aimed to investigate the effect of Pinacidil, a nonselective K_ATP_ channel opener, on diabetes-induced retinal gliosis and inflammation. Our results demonstrated that K_ATP_ channels are critically involved in diabetes-induced gliosis and microglial activation. Pinacidil could modulate microglial activation by upregulating Kir6.1 expression.

## Materials and Methods

### Animals

All animal experiments were conducted following the ARVO Statement for the Use of Animals in Ophthalmic and Vision Research and were approved by the Animal Ethics Committee of Central South University. Male Sprague Dawley (SD) rats, aged 10 weeks, weighing 250 ± 20 g, were obtained from the Animal Laboratory of Central South University and maintained in a 12-hour light/12-hour dark cycle at 22°C to 25°C and 55% to 60% humidity. Diabetes was induced using streptozotocin (65 mg/kg, STZ) via intraperitoneal (IP) injection. The blood glucose was measured (LifeScan, Milpitas, CA, USA) on day 3, weeks 1 and 2, and every month after STZ IP injection. The blood glucose levels maintained at >250 mg/dL were considered as diabetes.

### Intravitreal (IV) Injection

Four months after the onset of Diabetes (age-match rats were used as control), animals were anesthetized by IP injection of 2% *pelltobarbitalum natricum* (Sigma Aldrich, St. Louis, MO, USA) in a dose of 30mg/kg. Pupils were dilated using Tropicamide (Bausch & Lomb Freda Pharmaceutical Co. Ltd., Shandong, China) before the intervention. As previously described, IV injections were made at a distance of 1.0∼1.5mm posterior to the limbus with an angle pointing to the vitreous cavity, using a syringe with a 33G needle (Hamilton, Bonaduz, Switzerland) [31]. The pinacidil (85371-64-8; Sigma Aldrich, St. Louis, MO, USA) in 0.1% dimethyl sulfoxide (DMSO) were adjusted to the final concentrations 10, 50, or 100 µM (in a volume of 3 µL). For control, 0.1% DMSO in a volume of 3 µL was applied to the diabetic rats as a vehicle. Samples were harvested at 24 hours after IV injection. All animals were euthanized by decapitation before dissection.

### Cell Culture

Primary rat microglia were isolated and cultured following a published protocol.^18^ In brief, mixed retinal cells were obtained from P3-5 new-born SD rats using mechanical and chemical dissociation processes. Then, mixed retinal cells were seeded in T75 flasks with Dulbecco's modified Eagle's medium (DMEM)/F12 (1:1) containing 10% fetal bovine serum (FBS) and 1% penicillin streptomycin (PS) for two hours. Then fresh media was transferred to remove the floating cells. The medium containing recombined rat macrophage colony-stimulating factor (M-CSF; Novus Biological, Littleton, CO, USA) 100 ng/mL, 10% FBS 1% PS, was replaced every four to five days, until 80% confluence was achieved. To purify microglia, confluent cells were kept in DMEM/F12 containing 0.25% trypsin mixed in a 1:3 ratio for 35 to 50 minutes at 37°C as the “mild trypsinization” protocol as described,^19^ then the microglia cells remained and maintained with a medium containing M-CSF 100 ng/mL, 10% FBS, and 1% PS. The phenotype of retinal microglia was confirmed by CD11b and F4/80 staining (Supplementary Fig. S1). Primary microglial cells in the second or third passage were used in the experiments. Lipopolysaccharide (LPS) and IFN-γ–induced M1 cells and IL-4 induced M2 cells.

### Primary Human Retinal Müller Cell Culture

All experiments approaches were approved by the Ethics Committee of AIER Eye Group. Human eyecups were obtained from the AIER Eye Bank of Changsha (Changsha, China). Donors and their legally authorized representatives agreed to donate their eyeballs for scientific research. The retinal debris was carefully dissected and washed with PBS. After digested with trypsin-EDTA at 37°C for five minutes, the mixed retinal cells were seeded with DMEM containing 10% PBS and 0.1% PS, and maintained at 37°C, 5% CO_2_ for adherent. The cells were passaged three or four times and then used for experiments. Primary cells were routinely identified by the typical morphology (Supplementary Fig. S3A) and glutamine synthetase (GS) antibody (Supplementary Fig. S3B).

### Microglia-Müller Cell Co-Culture

**Figure 1.**
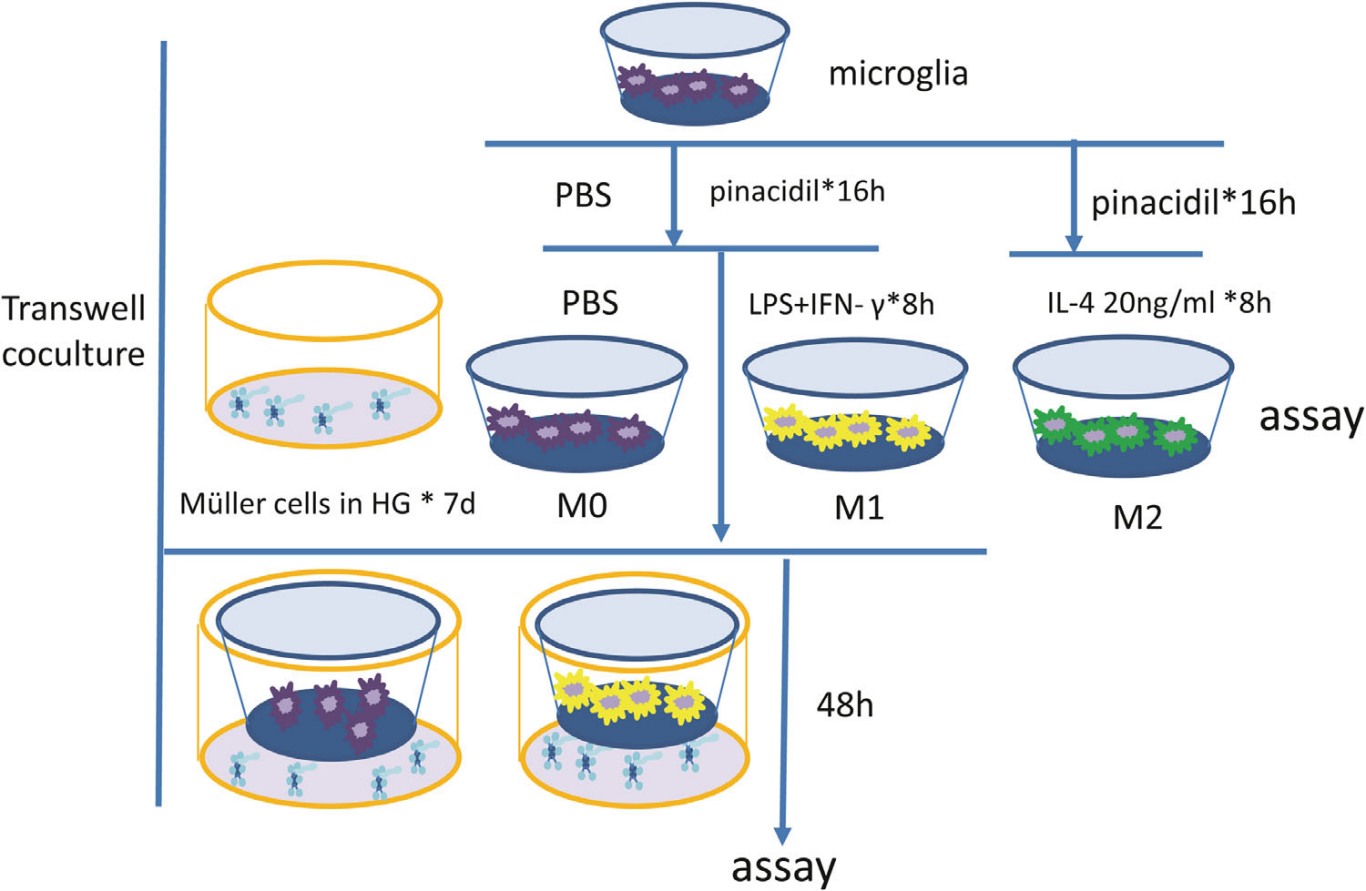
Diagram showing the microglia-Müller cells coculture system. The inserts containing nonactivated microglia (*M0*); activated microglia (*M1*) that were previously exposed to LPS 300 ng/mL and IFN-γ 20 ng/mL for eight hours; M2 cells that were cultured with IL-4 20 ng/mL for eight hours; pinacidil pretreated were applied 16 hours ahead of microglial phenotype induction.

The retinal Müller cells (rMc-1 cell line, obtained from Hunan Minghui Biological Technology Co., Ltd, Changsha, Hunan, China), or primary human retinal Müller cells were seeded and cultured in the bottom chamber of the Trans-well system (Corning, Inc., Corning, NY, USA) in DMEM (4.5 g/L glucose) with 5% FBS and 1% PS for seven days before the upper chamber with microglia was placed. The microglial cells were seeded into the upper chamber of Transwell plates permeable support membrane inserts (Corning, Inc.) at a density of 0.5 × 10^6^ cells/well in DMEM/F12 with 10% FBS and allowed to settle and grow for the next 24 hours. Then the microglia in the upper chamber were treated as desired for 16 hours. Before being cocultured, the cells were rinsed carefully with PBS to remove all the residual pretreated material. Then the microglial-Müller cell coculture was conducted for 48 hours in the following configurations, as shown in Figure 1.

### Immunofluorescence (IF) for Retinal Sections

Retinal frozen sections were prepared as described previously.^20^ Slices were incubated with the primary antibodies, as listed in Supplementary Table S1, at 4°C overnight, followed by secondary antibodies conjugated with FITC or Cy3 for one hour at room temperature and nuclei stained with DAPI. The sections were observed and imaged using a Nikon fluorescent microscope (Nikon ECLIPSETs2R; Nikon, Melville, NY, USA).

### Quantification of Iba1 Positive Nuclei

Microglia counts were performed on sections stained with anti-Iba1, a marker of macrophage as the previous literature.^21^ Briefly, Iba-1 positive nuclei in the outer plexiform layer (OPL), inner plexiform layer (IPL), and inner nuclear layer (INL) were assessed on the full length of retinal sections at the optic nerve level. The total counts of Iba-1 stained nuclei from each retina was the average of four sections and retinas from four rats per treatment group. Data were presented as mean ± SE.

### Quantitative PCR Analysis

Retinas or cells were lysed in Trizol (Life Technologies, Carlsbad, CA, USA). Total RNA was extracted with the Solarbio-R1200 RNA kit (Solarbio Life sciences, Inc., Beijing, China). The cDNA was synthesized with the PrimeScript RT Master Mix Perfect Real-Time kit (Takara, Japan). Quantitative real-time PCR (qRT-PCR) was performed using SYBR Mix (Roche Molecular Systems, Inc., Basel, Switzerland) following the manufacturer's instruction. The transcription level of target genes was detected with primer sequences listed in the Table and normalized by β-actin mRNA.

**Table.** Primers Applied in qRT-PCR

Target Gene	5-3	3-5
*CD86 rat*	ACGCAAGCTTATTTCAATGGG	GTTGTTCCTGTCAAAGCTCGT
*CD206 rat*	GCTGCTTCCAAACCTTTGAC	AGCTTCTCCACAGCCACAAT
*AQP4 rat*	TCGCCAAGTCCGTCTTCTACA	CCGTGGTGACTCCCAATCC
*kir6.1 rat*	AAGAGCATCATCCCGGAGGA	ATGTTCTTGTGTGCCAGGTTG
*kir4.1 rat*	ATTCTGGAAATCTTCATCACCG	GCACCCAATGAGGAGACTCTTAC
*Arg-1 rat*	CTCCAAGCCAAAGTCCTTAGAG	AGGAGCTGTCATTAGGGACATC
*TNF-α rat*	TGAGGACCAAGGAGGAAAGTATGT	CAGCAGGTGTCGTTGTTCAGG
*VEGF rat*	CAAAGCCAGCACATAGGAGAGA	CTATCTTTCTTTGGTCTGCATTCAC
*GFAP rat*	GCCCACCAAACTGGCTGAC	GTGCCGAGGTCCTGTGTGA
*Iba-1 rat*	AGGTGTCCAGTGGCTCCG	AGTTGGCTTCTGGTGTTCTTTG
*IL-1β rat*	ATGACCTGTTCTTTGAGGCTGAC	CGAGATGCTGCTGTGAGATTTG
*GS rat*	CGAAGACTTTGGGGTGATAGC	GGTCGTAGGCACGGATGTG
*CD86 mouse*	CAGCACGGACTTGAACAACC	CTCCACGGAAACAGCATCTGA
*CD11c mouse*	AGCGTGGAGAACTTTGATGCT	GGTAAGCGTCCCTCATGTCC
*GFAP human*	ATAGGCAGCCAGGTTGTTCT	GGGAGACTGAGGCAGGTATT

### Western Blotting Analysis

Retinas or cells were harvested and lysed in RIPA buffer (Life Technologies) containing protease and phosphatase inhibitor mini-tablets (No. 88668; Thermo Fisher Scientific, Waltham, MA, USA). Protein concentration was determined using a BCA protein assay kit (Solarbio Life sciences, Inc., Beijing, China). Denatured proteins (20 µg) were separated using Bolt Bis-Tris Plus polyacrylamide gel. After transfer, the polyvinylidene fluoride membranes were blotted with the primary antibodies at 4°C overnight, as listed in Supplementary Table S1. The β-actin was used as a loading control. After incubation for one hour with an appropriate Horseradish-peroxidase (HRP)-conjugated secondary antibody (Proteintech, Rosemont, IL, USA) at room temperature, the membrane was washed and developed with Pierce ECL Western Blotting-Substrate (Thermo Fisher Scientific). For the second or third probe, the membranes were stripped with Restore stripped buffer (Thermo Fisher Scientific) at room temperature for 30 minutes. Proteins were semiquantified by densitometry using Image J software (1.7, Java).

### Statistics

Statistical analysis was performed using GraphPad Prism (Version 6.0; GraphPad Software, La Jolla, CA, USA). The quantitative data were expressed as mean ± standard deviation. The one-way ANOVA followed by the Tukey-Kramer test was used for three or more groups comparison, and the paired Student *t*-test was applied for cell cultures between two groups. For animal experiments, the unpaired Student *t*-test was applied for two groups. *P* values < 0.05 were considered statistically significant. All experiments were repeated at least three times.

## Results

### Blood Glucose Levels and Body Weight in Diabetic Rats

Blood glucose and body weight were monitored at different times of the disease. After SZT IP injection, the rats showed significantly higher blood glucose levels (Supplementary Fig. S2A) and the loss of body weight (Supplementary Fig. S2B) compared to controls.

### Retinal Müller Cell Gliosis and Microglial Activation in Diabetic Rats

**Figure 2.**
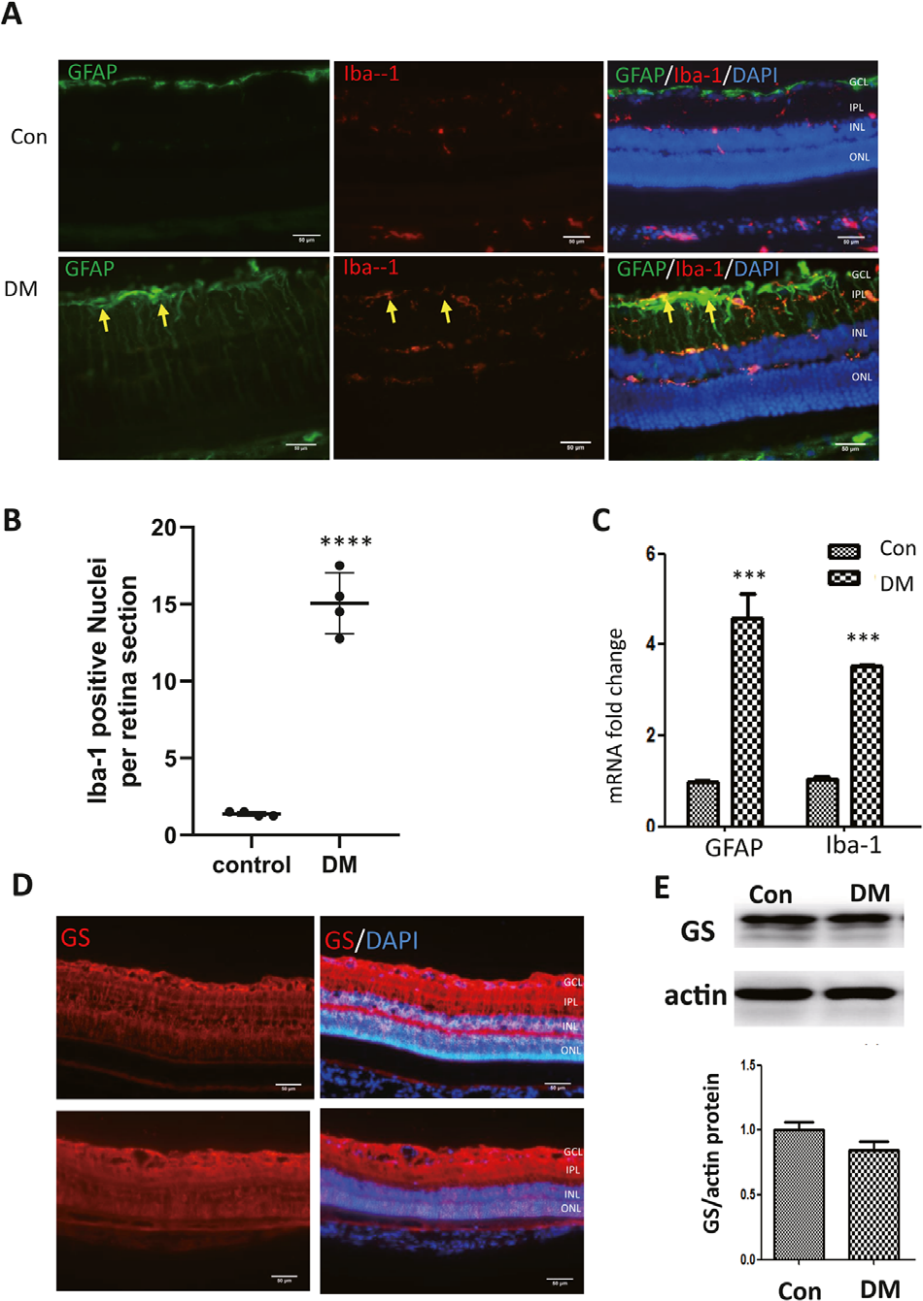
Detection of the Level GFAP and Iba-1 (IF, WB, qRT-PCR) in the 16-week DRs. (**A**) IF staining of GFAP and Iba-1 in the diabetic retina. GFAP positive cell crossed the whole retina. The *arrows* show the lesion with increased GFAP staining (*Scale bar*: 50 µm). (**B**) Quantification of Iba-1–positive nuclei (mean ± SM, two-way ANOVA, ****P < 0.0001). (**C**) The qRT-PCR result showed the transcription level of GFAP and Iba-1 in diabetic retinas (mean ± SD, unpaired *t*-test). (**D**) IF staining showed the GS in the diabetic retina was not changed (*Scale bar*: 50µm). (**E**) Western blot semi-quantification analysis the level of GS (mean ± SD, unpaired *t*-test, control (*Con*), diabetes mellitus (*DM*), *n* = 4; **P < 0.01; ***P < 0.001.

To evaluate the co-existence of Müller cell gliosis and microglial activation in diabetic rat retina, we conducted double-staining for the gliosis marker, glial fibrillary acidic protein (GFAP), and microglial marker, Iba-1. Sixteen weeks after diabetic induction, the GFAP was detected from the retinal nerve fiber/ganglion cell layer (GCL) to the outer limit membrane in diabetic retinas with discrete intense gliosis in the GCL (Fig. 2A). In normal rat retina, GFAP was only observed in the GCL (Fig. 2A). Iba-1+ cells were detected in the nerve fiber/GCL, IPL, and OPL, and the number of Iba-1+ nuclei was significantly increased in diabetic retinas (Figs. 2A and 2B). The qPCR results also showed that the expressions of GFAP and Iba-1 were increased in the diabetic retina (Fig. 2C). However, the GS level, Müller cell marker, was shown unchanged by IF (Fig. 2D) and Western Blot (WB) (Fig. 2E). Our results suggest that Müller cell gliosis and microglial activation were detected 16 weeks after diabetes.

### Pinacidil Alleviated Diabetes-Induced Müller Cell Gliosis and Microglial Activation

To determine whether the potassium channel regulates diabetes-induced Müller gliosis or microglial activation, pinacidil was applied through intravitreal injection at the sixteenth week after diabetes onset. We found 24 hours after pinacidil injection that the expression of GFAP and the number of Iba-1 positive cells were significantly reduced compared with DMSO vehicle controls (Figs. 3A and 3B). Furthermore, diabetes-mediated downregulation of Kir4.1 and upregulation of AQP4 was partially reversed by pinacidil (Fig. 3C). The effect of pinacidil on the GFAP and Iba-1 expression in the diabetic retina was further confirmed by Western blot (Fig. 3D). The diabetes-induced elevation of angiogenic and inflammatory factors VEGF (Fig. 3E) and TNF-α (Fig. 3F) were also suppressed by pinacidil.

**Figure 3.**
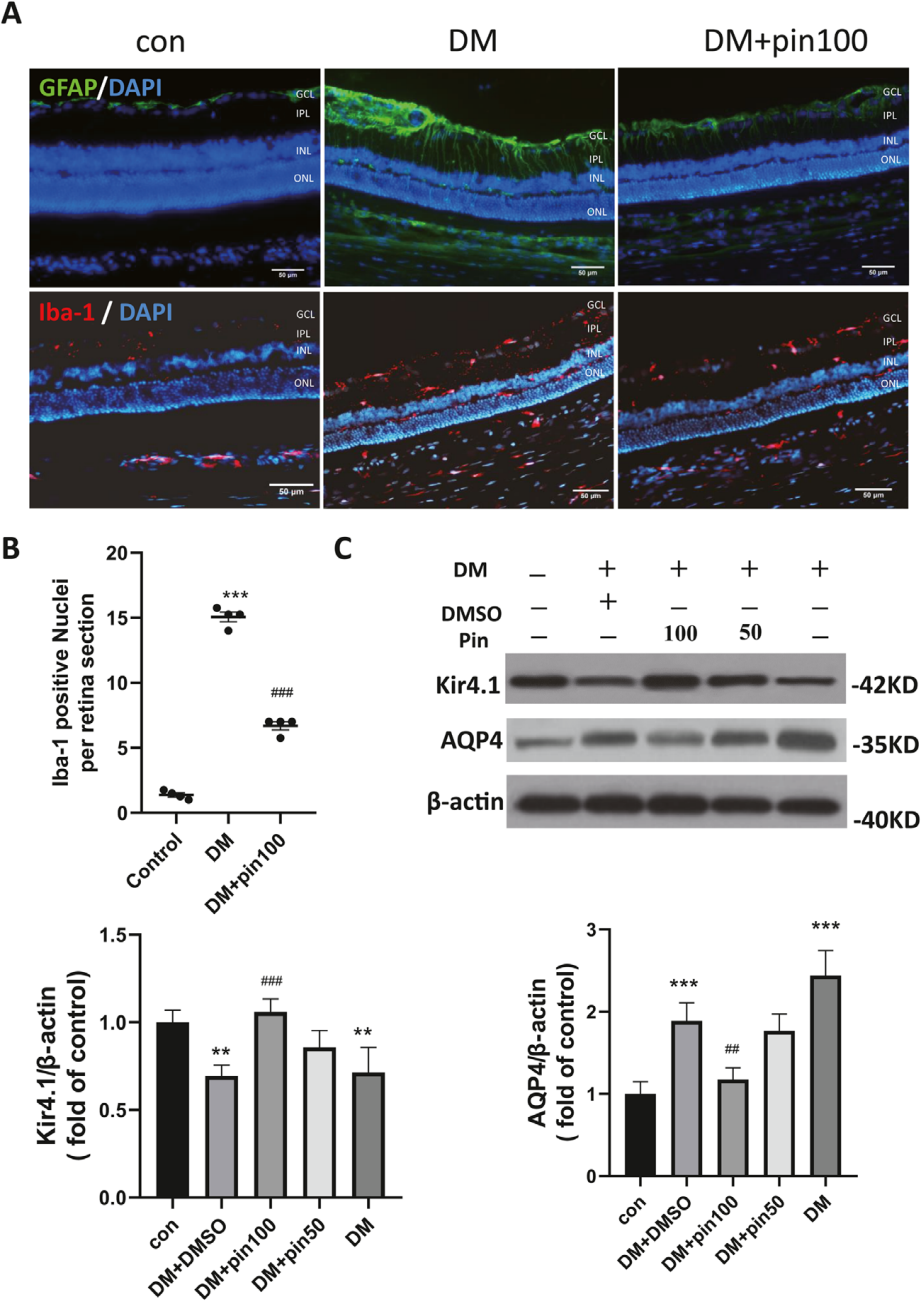
Pinacidil alleviated diabetes-induced Müller gliosis and microglial activation in rat retinas. (**A**) Presentative images of retinas double-stained with GFAP (*green*) and Iba-1 (*red*). (*Scale bar*: 50 µm). (**B**) Quantification of Iba-1 positive nuclei (mean ± SM, two-way ANOVA and Tukey's multiple comparisons test). (**C**) Western blot semi-quantification analysis the changes of Kir4.1, AQP4 in the rodent retinas (mean ± SD, one-way ANOVA and Tukey's multiple comparisons test). (**D**) Western blot semiquantification analysis the changes of GFAP, Iba-1, in the rodent retinas. (**E**) qRT-PCR examined the mRNA level of VEGF induced by diabetes and downregulated by pinacidil. (**F**) The mRNA level of TNF-α detected by qRT-PCR. (**G**) The mRNA level of CD86 and CD206 was examined in the diabetic retina. (**H**) Western blot semiquantification analysis the changes of CD86, GFAP, Iba1, Kir4.1, AQP4 in the normal rat retinas. (**I**) Presentative images of retinas double-stained with GFAP (***red***) and Iba-1 (*green*). (*Scale bar*: 50 µm). Pin100 = pinacidil 100 µM/3 µL, Pin50 = pinacidil 50 µM/3 µL. Data of WB and qRT-PCR represented as mean ± SD, *n* = 4. **P < 0.01; ***P < 0.001 vs. control group; ^##^*P* < 0.01; ^###^*P* < 0.001 vs DM. One-way ANOVA and Tukey's multiple comparisons test.

**Figure 3.**
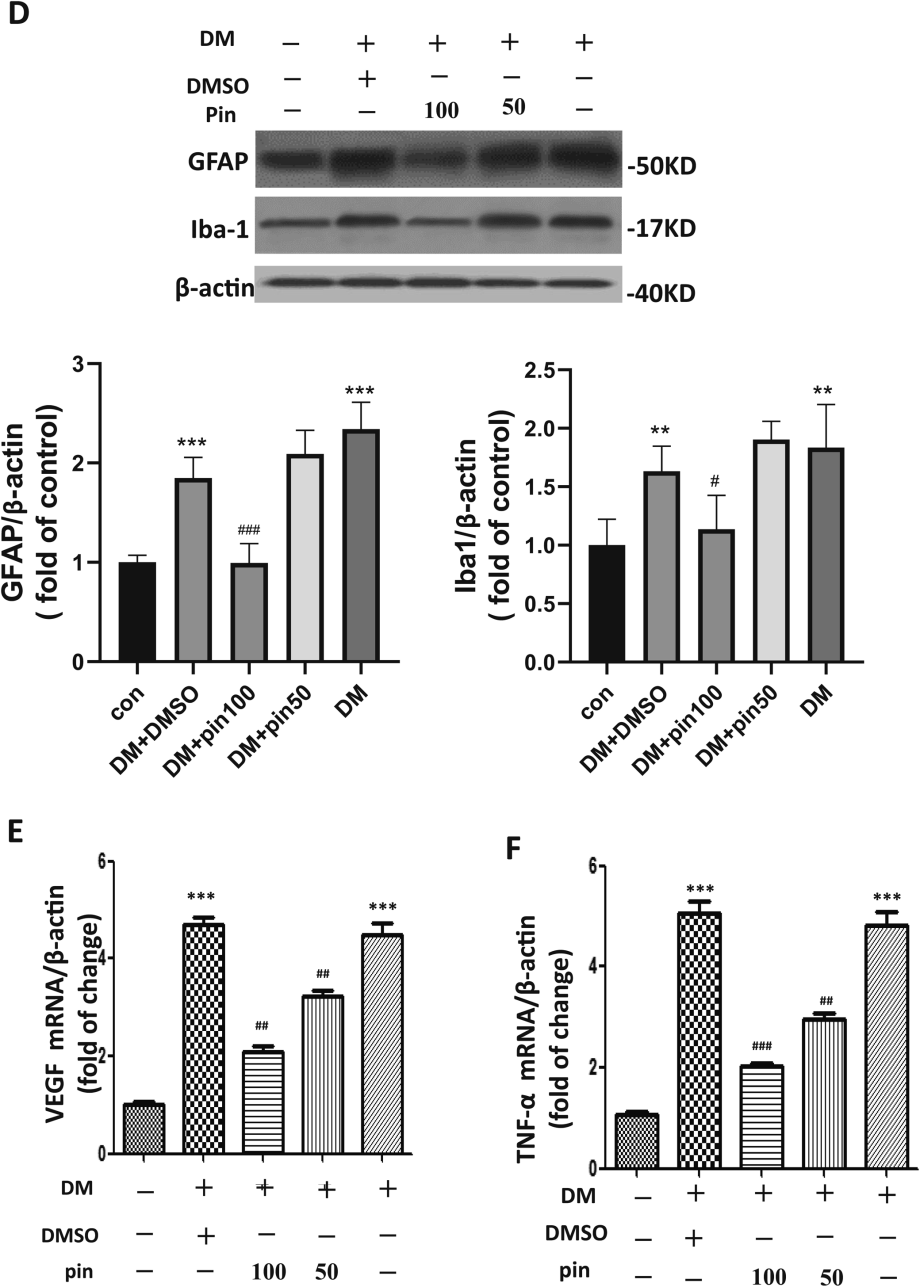
Continued

**Figure 3.**
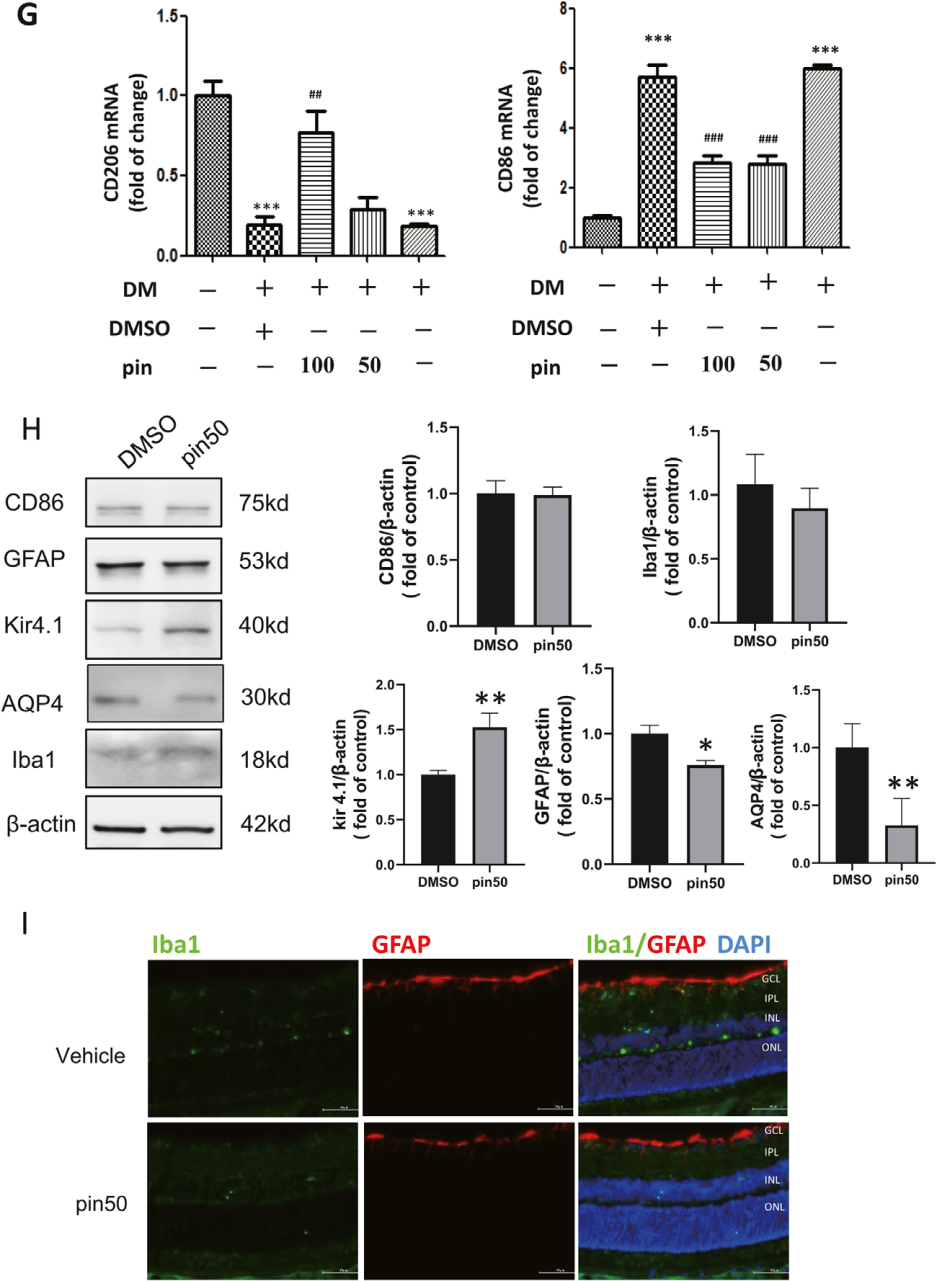
Continued

To further identify the active form of microglia, we analyzed the mRNA level of CD86, the marker of M1, and CD206, the marker of M2. The expression of CD86 mRNA was significantly increased by four times in diabetic retinas, whereas the CD206 mRNA level was significantly decreased in diabetic retina (Fig. 3G). The intravitreal administration of pinacidil significantly attenuated these alterations. Moreover, the intravitreal injection of pinacidil affected the expression of GFAP, Kir4.1, and AQP4 in normal rat retinas, but not the level of CD86 or Iba-1 (Fig. 3H). The retinal immunofluorescence showed, in normal retina, that pinacidil did not change the Iba-1 positive cells’ staining or locations (Fig. 3I). Our results suggest that intravitreal injection of pinacidil alleviated diabetes-induced retinal inflammation, Müller gliosis, and microglial activation by modulating the microglial phenotype transition.

### Pinacidil Attenuated Microglial Activation-Induced Müller Cells Gliosis and Kir4.1 Alteration

**Figure 4.**
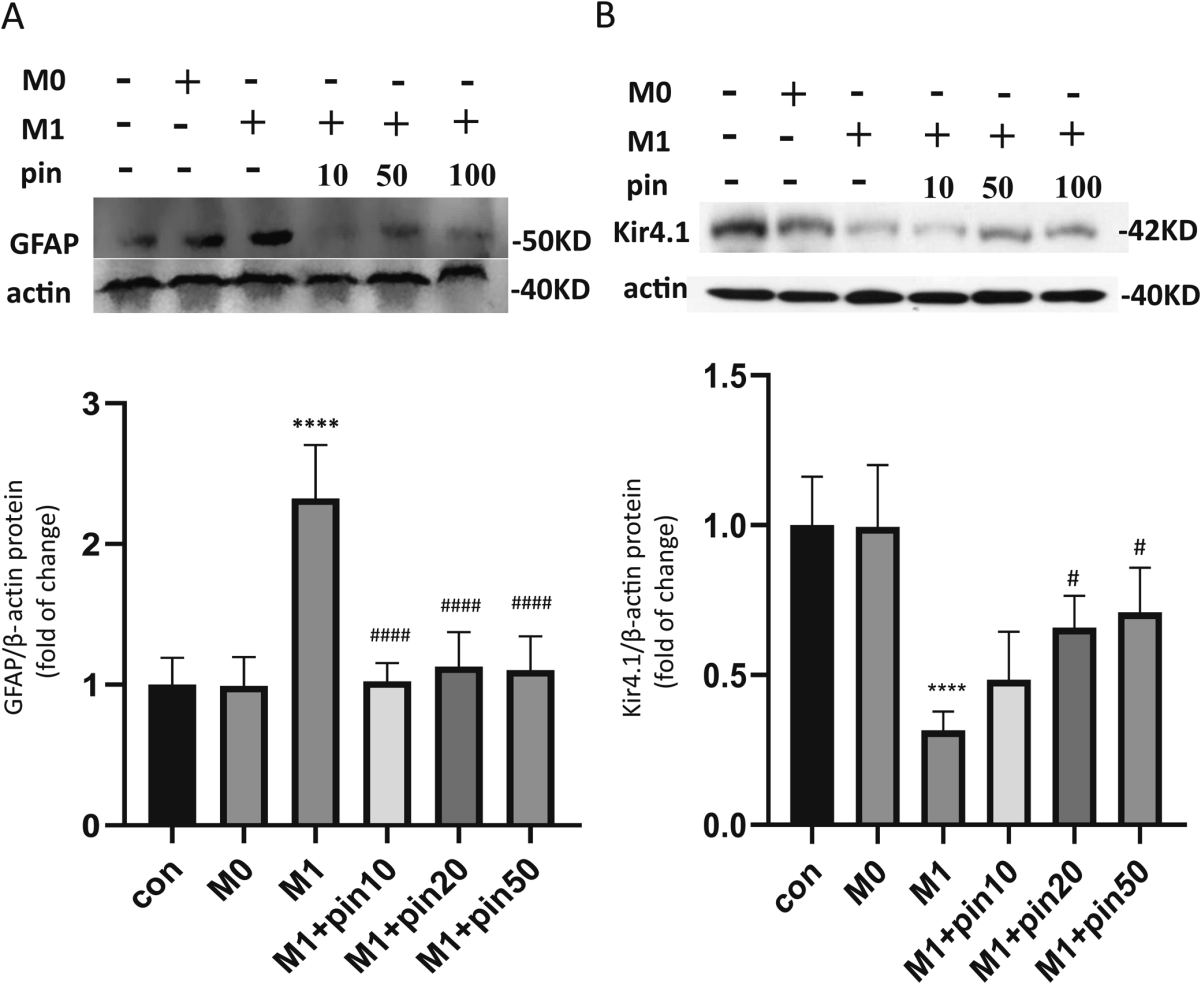
Pinacidil-treated microglia improved the GFAP, Kir4.1 expression in Müller cell in coculture model. (**A**) Western blot analysis of the change of GFAP in Müller cells, after cocultured with microglial cells with desired treatments. (**B**) Western blot analysis of the change of Kir4.1 in Müller cells, after cocultured with microglial cells with desired treatments. Data are expressed as mean ± SD, *n* = 4. ****P < 0.0001 when compared to M0 coculture group, ^#^*P* < 0.05; ^####^*P* < 0.0001 when compared with M1 coculture group. One-way ANOVA and Tukey's multiple comparisons test.

The coculture model and intervention were set up further to investigate the interaction between microglia and Müller cells, as shown in Figure 1. LPS 300ng/ml and IFN-γ 20 ng/mL were applied to stimulate the naïve microglia (M0) to transform into the proinflammatory phenotype, M1. Previously, we demonstrated that high glucose (HG, 25 mM) downregulated the Kir4.1 expression in cultured Müller cells.^12^ Then microglial cells were cocultured with rMC-1 pretreated high glucose. Coculturing with M0 microglia did not affect the expressions of GFAP or Kir4.1 in high glucose–pretreated rMC-1 cells (Fig. 4A). But M1 microglia significantly enhanced the levels of GFAP (Fig. 4A), whereas reduced the level of Kir4.1 (Fig. 4B) in rMC-1 cells. Interestingly, pinacidil pretreatment completely prevented M1 microglia-mediated GFAP upregulation (Fig. 4A) and partially rescued Kir4.1 downregulation in rMC-1 cells (Fig. 4B). These results suggest that pinacidil can regulate microglial activation affecting Müller gliosis and Kir4.1 expression.

Because the rMC-1 is an immortalized microglial cell line, we further confirm the effect of pinacidil in primary human retinal Müller cells (Supplementary Fig. S3A). Moreover, a microglial cell line, BV2 were used for upper chamber coculture. After intervention by LPS and IFN-γ, the mRNA level of the M1 marker, CD11c, and CD86 was induced, which could be reversed by pinacidil (Supplementary Figs. S4A and S4B). The mRNA level of GFAP in primary human retinal Müller cells was induced by M1, suppressed by pinacidil (Supplementary Fig. S4C). The change of protein level of GFAP and Kir 4.1 was similar to the finding in the rMC-1 cells (Supplementary Fig. S4D).

### Pinacidil Suppressed IL-1β and TNF-α Expression in LPS- and IFN-γ–Treated Microglia

**Figure 5.**
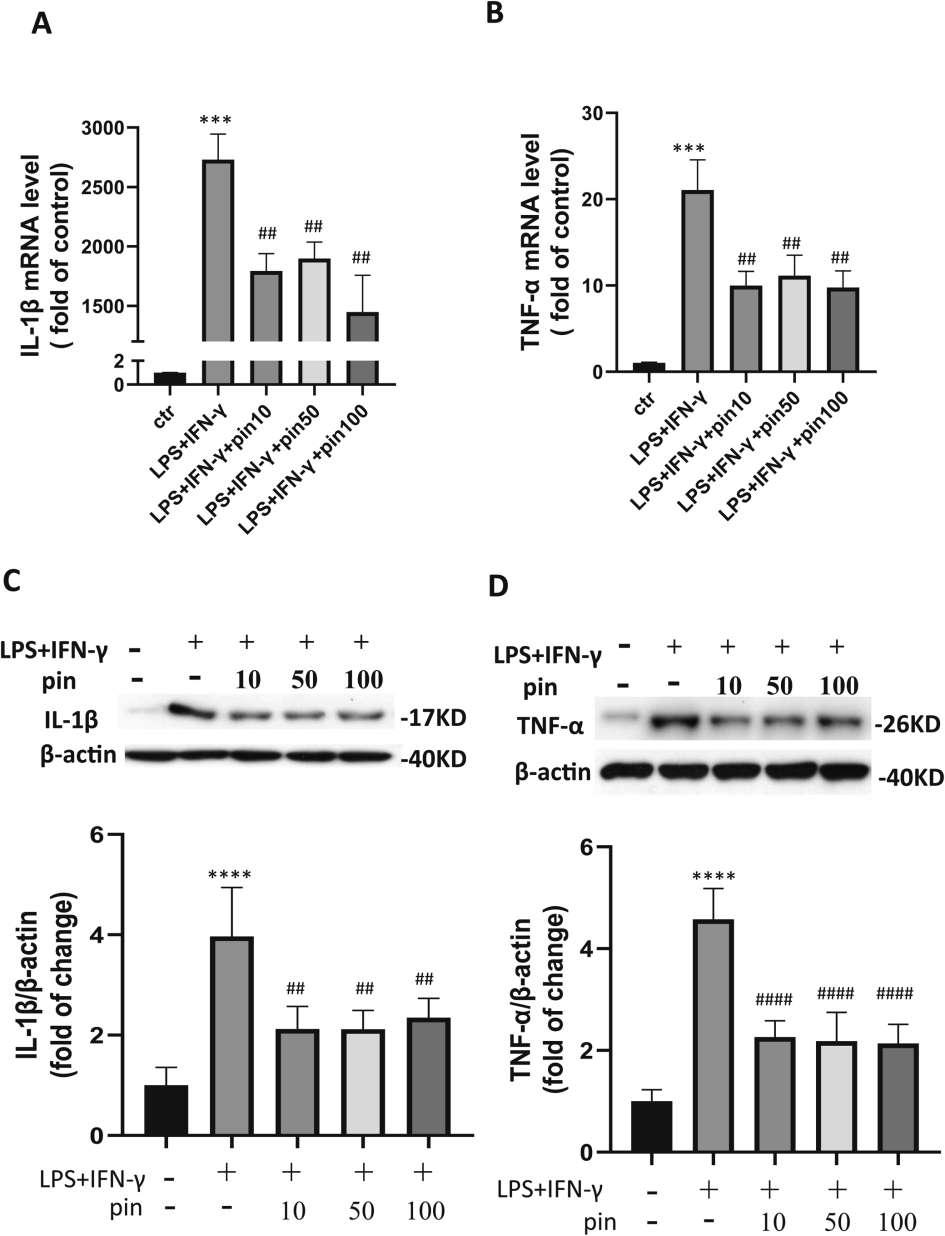
The effects of pinacidil on the proinflammatory phenotype transition of microglia. (**A** and **B**) The transcription level of IL-1β (**A**) and TNF-α (**B**) was detected by qRT-PCR. (**C** and **D**) Western blot semiquantification analysis revealed upregulation of IL-1β (**C**) and TNFα (**D**) in M1 cells but suppressed by pinacidil pretreatment. Data represented as mean ± SD, n = 4. ***P < 0.001, ****P < 0.0001 vs. control group (without treated), ^##^*P* < 0.01, ^####^*P* < 0.0001 vs. LPS and IFN-γ (+) and Pin (−) group, one-way ANOVA and Tukey's multiple comparisons test.

To further decipher the effect of pinacidil on microglia, we examined the proinflammatory cytokine levels in M1 microglia treated with different pinacidil concentrations. As shown in Figure 5, the mRNA transcription levels (Fig. 5A) and protein expression levels of IL-1β (Fig. 5B) and TNF-α (Fig. 5C) were significantly increased in M1 microglia, and the increment was significantly inhibited by pinacidil treatment (Figs. 5A–5C).

### Pinacidil Induced Kir6.1 Expression and Enhanced the M2 Transition

**Figure 6.**
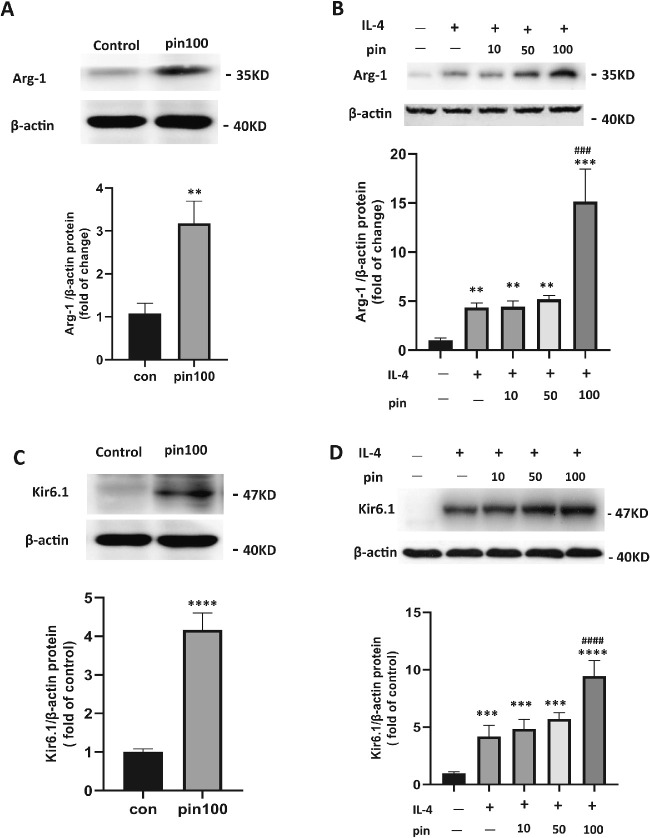
The effects of pinacidil on the proinflammatory phenotype transition of microglia and Kir6.1 expression. (**A**) Western blot semiquantification analysis indicated that pinacidil upregulated the expression of Arg-1 in microglial cells without IL-4. (**B**) Arg-1 was induced by IL-4 treatment. Pinacidil enhanced the effect of IL-4 on the expression of Arg-1. (**C**) Western blot semiquantification analysis indicated that pinacidil upregulated the expression of Kir6.1 in microglial cells. (**B**) Kir6.1 was induced by IL-4 treatment, which was enhanced by Pinacidil. Data represented as mean ± SD, *n* = 4. **P < 0.01; ***P < 0.001; ****P < 0.0001 vs. negative group; ^###^*P* < 0.001; ^####^*P* < 0.0001 vs. IL-4 intervention group, one-way ANOVA and Tukey's multiple comparisons test, and paired Student *t*-test for two groups comparison.

A previous investigation has shown that Kir6.1 is expressed explicitly in brain microglia, which plays a role in the development of the central nervous system degenerative disease.^15^ To elucidate the mechanism in which pinacidil affects microglia, IL-4 was used to induce M2, anti-inflammatory microglial phenotype. Because Arg-1–positive microglia showed an anti-inflammation effect in neuron system diseases and are considered one of the markers for M2 transition, the level of Arg-1 was evaluated. The level Arg-1 (Figs. 6A and 6B, lane 2) and Kir6.1 (Figs. 6C and 6D, lane 2) were significantly upregulated by pinacidil with or without IL-4. Pinacidil pretreatment further enhanced IL-4–induced upregulation of Kir6.1 and Arg-1 expression (Figs. 6B and 6D). Our results suggest that the pinacidil promotes microglial M2 differentiation and Kir6.1 channel expression.

## Discussion

Pinacidil is a nonselective ATP-sensitive potassium (K_ATP_) channel opener, including the plasma membrane K_ATP_ (pmK_ATP_) and mitochondria K_ATP_ (mitoK_ATP_) channels, relaxing the cardiovascular smooth muscles. It has been widely used to treat hypertension for more than two decades. Previously, Ishizaki et al.^22^ demonstrated that K_ATP_ channels are located in the retinal microvasculature, and the pinacidil-induced K^+^ current plays a role in the regulation of retinal blood flow, which is involved in the development of diabetic retinopathy. Interestingly, our recent investigation showed that the downregulation of Kir4.1 in Müller cells plays a vital role in diabetes-induced retinal inflammation, which can be reversed by systemic application of pinacidil.^12^ In the present study, we show that proinflammatory microglia could promote Müller cell gliosis in diabetes, and pinacidil could directly modulate microglial phenotype, in turn reducing the retinal inflammation and gliosis. It is suggested that pinacidil may be repurposed in DR treatments.

Numerous studies demonstrated that chronic low-grade inflammation exists in diabetic rodent retinas and suggests that inflammation contributes to DR and DME.^23^ In diabetic rodent retinas, the microglial transition between these two phenotypes was deeply involved in diabetes-induced retinal inflammation, as has been reported by Arroba et al.^24^ In the early stages of DR^2^, M2 response balances M1, ameliorates inflammation, and delays disease progression. As the disease progression, the M1 response is maintained, whereas that of M2 declined. Furthermore, retinal Müller glia is also responsible for developing DR by releasing inflammatory cytokines and gliosis activation. Our recent study showed that pinacidil suppressed LPS and IFN-γ-induced CD11c and CD86, markers of M1 microglial subtype, in cultured microglial cells. We also showed that pinacidil increased the expression of arginase-1, which major existing in the M2 subtype in the murine retina. These results suggested that M0 to M1 transitions were attenuated by pinacidil, and the M2 marker was also induced. However, we cannot rule out the possibility of the M1 subtype returning to a resting morphology M0.

However, because the morphologic approach cannot identify the M1 and M2 phenotype differences, we did not investigate morphology to define microglial activation. Alternatively, we quantified the Iba1 positive nuclei in the retinal sections, as described by Wang et al.,^17^ who demonstrated that the microglial cell density and Iba-1 immunopositivity were increased in retinal inflammation. Moreover, qPCR of cell marker M1 (CD11c) and M2 (CD86) was applied in this study.

On the other hand, the evidence demonstrating the crosstalk mechanism between microglia–Müller glia in diabetic inflammation was inadequate.^2^^,^^25^^,^^26^ Consistently, we detected the disturbed balance between M1 and M2 in rat retina in diabetes and Müller cell gliosis. Moreover, we revealed that these alterations were dramatically attenuated by pinacidil. These findings demonstrated in vitro that pinacidil inhibited the LPS-induced M1 polarization and suppressed TNF-α and IL-1β production. Pinacidil on its own can increase the expression of M2 marker Arg-1 and further enhance IL-4–induced M2 polarization. Our results suggest that the K_ATP_ channel opener alleviates the microglial-associated inflammation by modulating microglial polarization. The induction of M2-type microglia by pinacidil reduced high glucose–induced Müller cell gliosis, strong evidence of how microglia-Müller cell crosstalk in DR K_ATP_ channel may involve in this mechanism.

**Figure 7.**
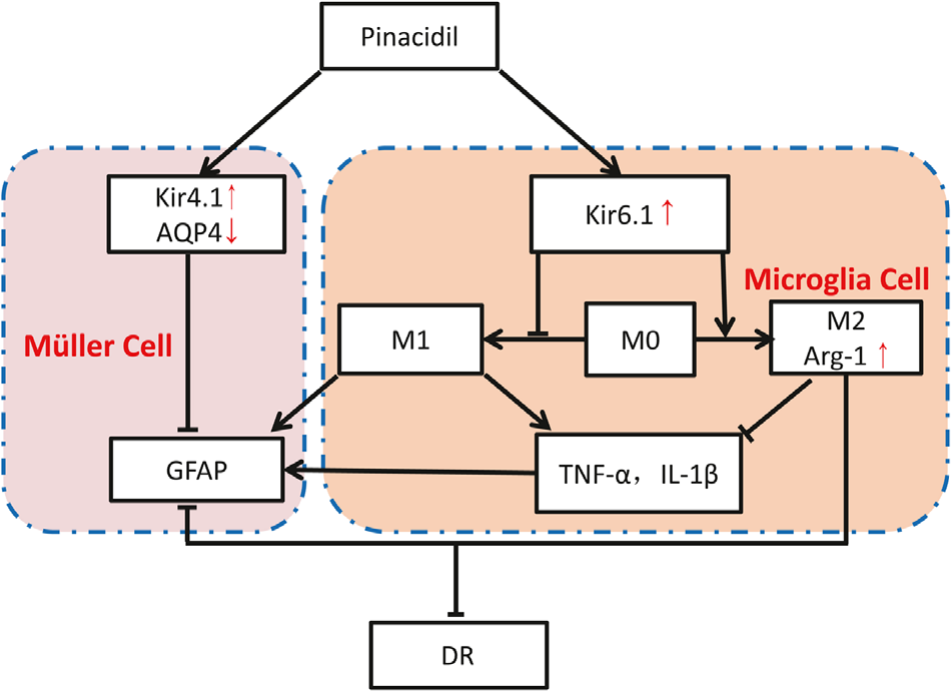
Schematic summary of the potential mechanism of pinacidil involved in the treatment of DR. Pinacidil restrains the activation of the proinflammatory phenotype of microglia and promotes anti-inflammatory phenotype by enhancing the expression of Kir6.1, which then inhibits the Müller gliosis and augments the expression of Kir4.1 in Müller cells. On the other hand, pinacidil directly acts on the Kir4.1 in Müller cells and suppresses Müller gliosis. Pinacidil regulates the retinal inflammation dually by targeting microglia-Müller glia interactions in DR.

Nguyen and colleagues^27^^,^^28^ have shown that the microglial potassium channel expression is very plastic and possibly has species differences. We found that the K_ATP_ channel is also plastic and is linked to their phenotype in retinal microglia. We show that Kir6.1 was upregulated in IL-4–induced M2 microglia alongside Arg-1, which could be further enhanced by pinacidil. The expression of Arg-1 is in parallel with the level of Kir6.1. A previous study has shown that overexpression of Kir6.1 resulted in switching microglia from the detrimental M1 phenotype to the beneficial M2 phenotype via p38 MAPK–NF-κB signaling pathway inhibition. This mechanism was implicated in the chronic inflammation of Parkinson's disease.^15^ The same group also demonstrated that Kir6.1 knockdown aggravates cerebral ischemia/reperfusion-induced neural injury by augmenting reactive glia and inflammatory responses.^29^ These results suggest that, in diabetes, M1-type proinflammatory microglia facilitate Müller gliosis and reduce Kir4.1 expression. Together, pinacidil may suppress gliosis and inflammation by upregulating the Kir4.1 expression in retinal Müller cells and modulating M1 and M2's transition ratio by upregulating Kir6.1 in retinal microglial cells (Fig. 7). However, in the present study, the regulation mechanisms of how pinacidil affect the expression of Kir4.1 and Kir6.1 are still elusive. Future studies should be conducted to reveal the underlining mechanisms.

## Conclusions

Our results suggest that potassium channels are involved in diabetes-induced gliosis and microglial activation. The K_ATP_ opener, pinacidil, can reduce microglial activation by upregulating Kir6.1 expression, which then inhibited the Müller gliosis and augmented the expression of Kir4.1 in Müller cells. These findings provide evidence for pinacidil regulating the microglia-Müller glia interactions in the diabetic retina.

## Supplementary Material

Supplement 1

Supplement 2
