# Supplementary material for: KATP Opener Attenuates Diabetic-Induced Müller Gliosis and Inflammation by Modulating Kir6.1 in Microglia
Source: Invest Ophthalmol Vis Sci. 2021 Feb 1;62(2):3. doi: 10.1167/iovs.62.2.3 (PMC7862730; doi:10.1167/iovs.62.2.3)
Supplement: Supplement 1 [file iovs-62-2-3_s001.pdf]

Supplement figure 1

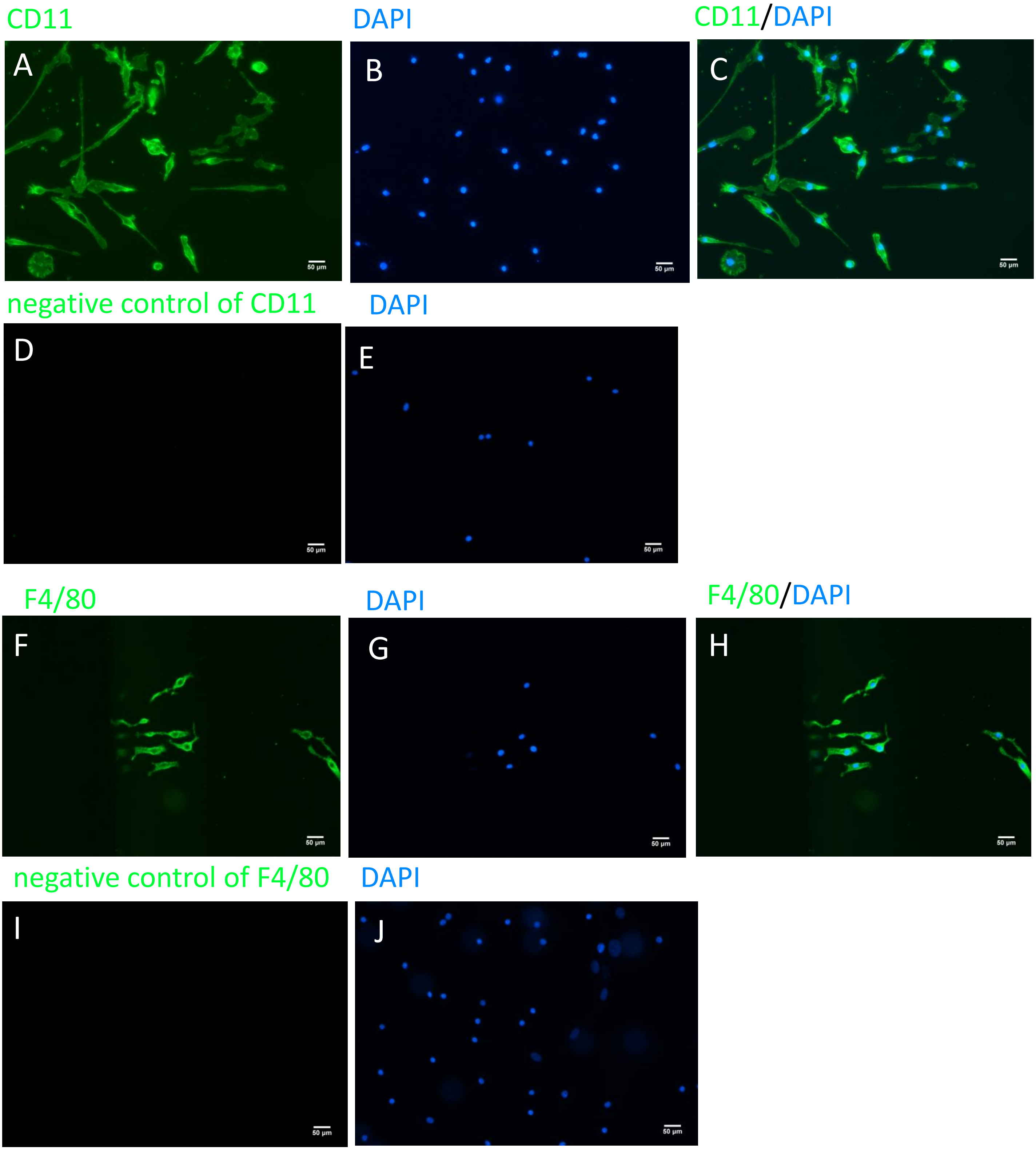

Cells were stained with CD11 or F4/80 for identification. (A~C). Cell were stained with microphage marker CD11, nuclei were stained with DAPI, then two images were merged as C. (D~E). Negative control were done with the same secondary antibody without CD11. (F~H). Cell were stained with microphage marker F4/80, nuclei were stained with DAPI , then two images were merged as H. (I~J). Negative control were done with the same secondary antibody without F4/80. Scale bars, 50μm.

Supplement figure 2

A

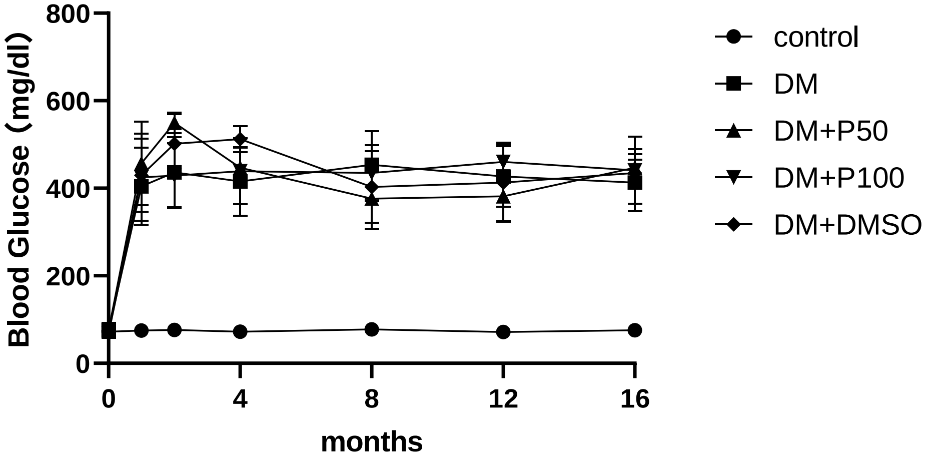

B

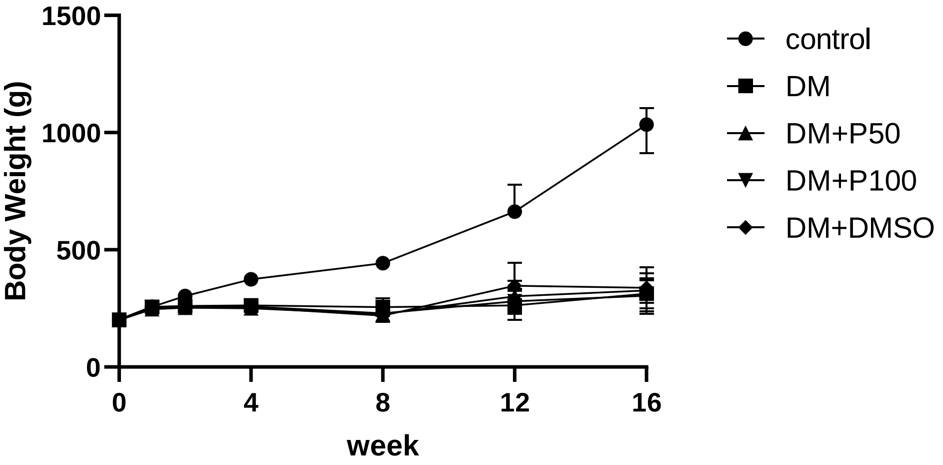

Blood glucose (A) and body weight (B) were monitored routinely in each group. Blood glucose was increased after STZ IP injection, and maintain over 15mmol/l until 16 weeks in DM groups.

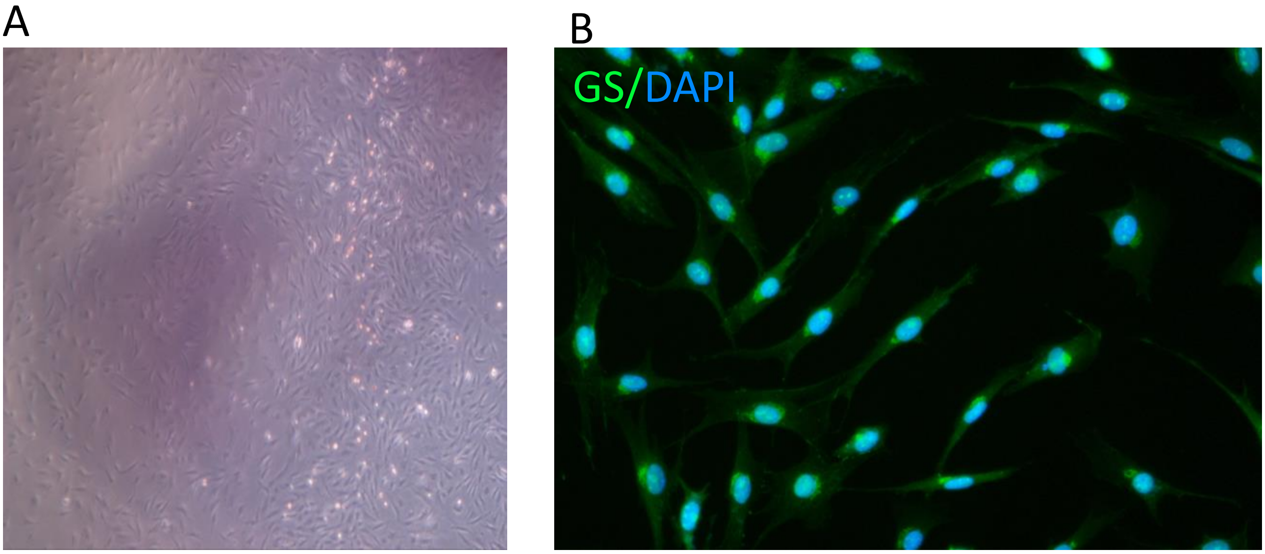

Identification of primary human retinal Müller Cells.

A. Bright flied image of the primary human retinal Müller Cells. B. Cell were stained with Müller Cells marker GS (green), nuclei were stained with DAPI (blue).

Supplement figure 4

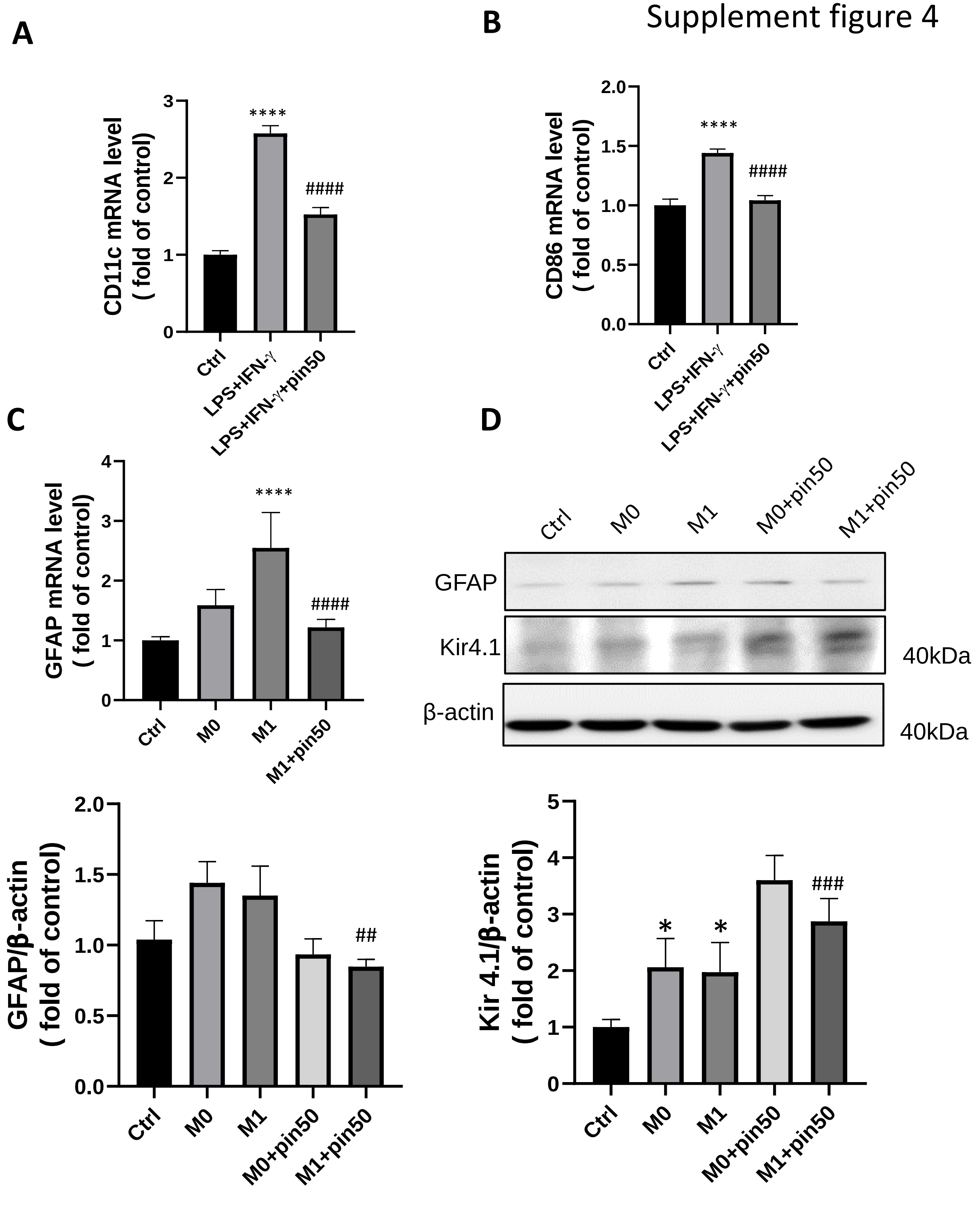

Supplement figure 4. The mRNA level of M1 marker, CD11c (A) and CD86 (B) was induced by LPS and IFN-γ the in BV2 microglial cells. C. The mRNA level of GFAP in primary human retinal Müller cells was induced by M1 and were suppressed by pinacidil. D. Kir 4.1 was upregulated and GFAP was downregulated by pinacidil when compared to M1 group. Data are expressed as mean ± SD, n=3~4. *P< 0.05, ***P< 0.001, ****P< 0.0001 when compared to control group, # P<0.05, ##P < 0.01, ###P< 0.001, #### P< 0.0001 when compared with M1 coculture group. One-way ANOVA and Tukey's multiple comparisons test.
