# Supplementary material for: KATP Opener Attenuates Diabetic-Induced Müller Gliosis and Inflammation by Modulating Kir6.1 in Microglia
Source: Invest Ophthalmol Vis Sci. 2021 Feb 1;62(2):3. doi: 10.1167/iovs.62.2.3 (PMC7862730; doi:10.1167/iovs.62.2.3)
Supplement: Supplement 2 [file iovs-62-2-3_s002.pdf]

Table 1S. Antibodies applied in immunostaining or western blotting.

Antibody	*IF Dilutions*	*WB Dilutions*	*Catalog No.*	*Provider*
anti- GFAP	1:100		MA5-12023	Thermo Fisher
anti-β-actin		1:5000	60008-1-Ig	Proteintech
anti- glutathione synthase (GS)	1:200	1:1000	ab49873	Abcam
anti- Iba-1	1:200	1:500	ab15690	Abcam
anti- Arginase-1		1:1000	NBP1-32731	NOVUS
anti-GFAP		1:500	bs-0199R	BIOSS
anti-TNFα		1:500	ab6671	Abcam
anti-Kir4.1		1:1000	ab192406,	Abcam
anti- AQP4		1:500	ab46182	Abcam
anti- Kir6.1		1:500	ab241996	Abcam
anti- IL-1β		0.2 µg /ml	ab9722	Abcam
FITC-anti-mouse	1:500		A16067	Life Technologies
Cy3-anti-rabbit	1:500		A16177	Life Technologies
